# Mr. Lee on the Medical Institutions of France, Germany, and Italy

**Published:** 1836-01-01

**Authors:** 


### Observations on the Medical Institutions and Practice of France, Germany, and Italy, &c. &c. By *Edwin Lee*, M.R.C.S. formerly House-Surgeon of St. George's Hospital. Octavo, Churchill, October, 1835.

If knowledge were increased in proportion as medical men run to and fro, on the Continent, medicine would now be at a very high pitch in this country. English physicians and surgeons have not, we believe, improved *directly*, in their *practice*, by studying on the Continent. But they may have improved *indirectly* by acquiring more accurate and comprehensive knowledge of pathology. But even this advantage has been somewhat over-rated. Morbid anatomy is not precisely the same in Germany and in England. This may appear a startling proposition, but it is a fact. If two men, of the same age, and, as nearly as possible, of similar constitutions, die in Berlin and London, of pneumonia, the same appearances will not be presented in both bodies. The pathological characters in the German body will be on a larger scale, or rather more intense in degree. The one will be an exaggeration, or kind of caricature of the other. And why? Because the pathology is modified by the treatment, which is so much more active in this country than on the Continent. Any one may become convinced of this without crossing the Channel. Compare continental delineations of morbid anatomy, especially where inflammation is concerned, with the appearances on dissection here, and you will conclude that our foreign neighbours have magnified or exaggerated. Still, it is but fair to acknowledge that our pathology has been much improved by our intercourse with France, Germany, and Italy, since the termination of the war. The advantages have been reciprocal; for our Gallic brethren in particular are rapidly improving in the energy of their practice—partly from observing the treatment of disease in this country, and partly from the criticisms which they read on their own practice.

Mr. Lee is known to the professional public by his "Notes on Italy and Rhenish Germany," published a few years ago. The present is a republication, enlarged, of the same work, including several places in Germany, not noticed in the first work. It will be a very useful companion for those medical gentlemen who are about to seek information on the Continent. It is not susceptible of regular analysis; but we shall cull from it a few matters that may be interesting to fire-side travellers at home. The following short extract may be useful in high quarters. Speaking of the French hospitals, Mr. Lee observes—

"Hospital physicians and surgeons are usually chosen from among the members of the central office. They are in general exceedingly regular in their attendance, make their visits at an early hour every morning, and during the visit avail themselves of every opportunity of imparting clinical information to the students. Patients are minutely examined, and the daily progress of their case taken in writing by a clinical clerk attached to each professor. After the visit the professors of the faculty, and some others, give clinical lectures, and see out-patients." 2.

We do not suppose that the governors of our hospitals will ever give up their privileges—their "vested rights"—and allow the election of their officers to be taken out of their own hands. The polity of the Parisian institutions is so totally different from that of ours, that the same discipline can never obtain in both.

It is well known that the members of the medical profession, on the Continent, do not hold so high a degree of consideration in society as they do in England—and the scale of remuneration is much lower. How are we to account for this? The following is Mr. Lee's explanation.

"The trifling expense of the course of study allows admission to many of the poorer class of individuals, who would be excluded in England by the expenses attendant on their education. Many students exist upon one thousand francs, or even less, per annum, and in case of sickness are necessitated to become inmates of the hospitals, where they find more attention and advantages than could be expected in their comfortless apartments." 16.

We have often touched on this subject before, and always with reluctance; but the good of our profession, and no personal motives, induces to advert to it again. No one will accuse, or even suspect us, of a leaning to the high aristocracy, either within or without the pale of the profession; but a daily observation of the miseries and degradations to which medical men are reduced, by the redundancy of their ranks, forces us to raise our voice against all *incentives* to enter the faculty of physic. If, indeed we wish to see the practitioners of the healing art degraded below the rank of the pettiest shop-keepers, we shall then advocate the principle of making every man a DOCTOR, who can afford to expend a couple of hundred pounds on his professional education. The consequences of such facilities of introduction to a liberal profession will be disastrous in the extreme. The multitudes who will aspire to the practice of physic will involve the whole in ridicule and contempt. We will give an illustration which has just presented itself to our notice. A poor clergyman whom we recommended to take a tour up the Rhine and through Switzerland, was taken ill in a town on the Continent, and sent for a medical man who was a physician, surgeon, and apothecary. This was on the 5th of September, 1835, and he was discharged cured, on the 17th of the same month. The clergyman was an exceedingly intelligent gentleman, and he shewed us the whole plan of treatment. We have no hesitation in declaring that it was the very worst that could possibly have been adopted. The wonder is, that the poor patient survived the disease and the *mala praxis* combined! And what was the bill for these twelve days' attendance? Only 30 Napoleons! Nearly 2*l.* 10*s.* per day!! The very inn-keeper cried, like a child, when he saw this bill, and declared that such a medical practitioner was a national disgrace! When a remonstrance took place, this cheaply educated doctor of medicine, surgery, and pharmacy alleged, in extenuation of his *modest* bill, that he acted as the physician, *nurse,* and *servant* of the patient!! When cheap education enables such people to come into the ranks not by hundreds, but by thousands, we may easily anticipate a *levelling downwards* of the profession, till the continental rank is reached.*

We do not, indeed, advocate a scale of high fees to teachers and hospitals, but a long and laborious course of study, which will answer the double purpose of checking the influx, and bettering the quality of practitioners. There will be no danger of scarcity in numbers, however high we raise the standard of education. It will, therefore, be madness not to strain the curriculum to the very utmost latitude, when a new code of medical legislation comes under the consideration of Parliament.

---

* By an extract from the poor-law commissioners report, in No. 631 of the Lancet, we grieve to see instances, little inferior in turpitude to the foregoing. It is stated, that medical men have been in the habit of bribing the inferior parish officers to search out for extra-parochial paupers, for attendance on which, there was no limit to the amount—the charge made being levied on another parish! What, indeed, must be the state of our profession, when parish officers can get plenty of medical men to contract for medicines and attendance at the rate of two or three shillings per head per annum!!

Most medical elections are decided, on the Continent, by the *Concours*, a system greatly superior to the English mode of election, but, for the reasons before given, we can have little hope of such a procedure among the purse-proud and bull-headed governors of British hospitals.

Mr. Lee has found that the number of foreign practitioners, who now adhere to the purely " expectant measures in the treatment of disease," is very limited. This has resulted, in a great measure, from familiarity with English practice.

" The majority, however, appear to be reverting to the principles of the Médecine Hippocratique, which, less disposed to generalize and to consider inflammation present in most diseases, adopts less energetic measures, the practice being regulated by the actual condition of the symptoms in individual cases. The hospital practice of men professing opposite opinions is not, however, so different as might be expected from a perusal of their works. A pharmaceutical combination of drugs is not frequently employed, the remedies being mostly of a simple nature, and except the difference in regard to sanguineous depletion; tisanes, decoctions of simple herbs, mucilaginous and sweetened beverages, joined to tepid baths and enemata, are the medical means mostly used in the great majority of cases." 17.

Those who are of the Broussaian school—and the number is very considerable—proscribe purgatives in fevers, and, consequently, deprive themselves of one of the most effective weapons in warring with the disease! Mr. Lee is surprised that the diffusion of pathological knowledge in France has not led to more energetic practice, and endeavours to account for the circumstance in the following manner.

" This may, however, in my opinion, be accounted for by two circumstances; —first, that post-mortem lesions, the effects of disease, are often mistaken for its cause, and consequently the treatment must be less successful than one based merely upon the observation of symptoms; and secondly, that a routine line of practice is adopted against diseases; sufficient attention not being paid to the various modifications the same disease may assume in different persons, the patient's constitution, strength, and other peculiarities of his case, not being sufficiently taken into account." 21.

If they mistake effects for causes, which we fear that a great many of them do, it does not speak much for their sagacity or reasoning powers. We do not deny that active remedies are often too freely used in this country; but the waste of human life resulting from the inert practice of the Continent is a far greater evil. Mr. Lee is rather mistaken in supposing that auscultation and percussion are at such a low ebb in this country. We have the means of knowing, that these important means and modes of investigation are becoming almost general in this country. The following passage will shew that the Continent is not the best school for the young *surgical* student.

" In several points connected with the treatment of surgical disease, there exists a material difference between England and the Continent. The most prominent of these consists in the absence of internal treatment in most surgical cases, by which surgery is reduced to little more than the application of dressings and the performance of operations. In France and Italy, scarcely any medicine is given in surgical diseases, the means of relief being principally restricted to rest, the general and local abstraction of blood, local applications, including counter-irritants, and, lastly, operations; which are often performed in cases where their necessity would be obviated in England by the timely adoption of measures influencing the progress of the local disease, by their operation on the general system." 24.

This striking defect vitiates the whole of the Continental surgical practice, and not only so, but it often renders their writings useless—sometimes even injurious to British practitioners. Medical men of clinical experience, in this country, have long been aware of this circumstance, and hence the very feeble avidity with which Continental writings, especially surgical, have been and will be received in Great Britain.

"With respect to mercury, its action on the capillary system of vessels, and consequent effects in controlling inflammatory disease, do not appear to be known, or, if known, are not appreciated by continental practitioners. Calomel is generally considered merely as a purgative, and is occasionally administered as such. One writer in a popular work even says, that this medicine is almost entirely inert, and may be given in large quantities with impunity."* 25.

Mr. Lee remarks, that his observation in Continental hospitals induces him to believe, that numbers die annually there after accidents and operations, from starvation and constipated bowels, who, in this country, would certainly be saved by proper treatment. He has seen repeatedly patients allowed to go six, eight, and ten days, without ever being relieved in their bowels !!

They have, at length, abandoned the practice, or rather the theory, of healing wounds by granulation instead of adhesion—but it is only in doctrine, for, what with starvation and awkward dressing, adhesion by the first intention is a rare occurrence.

We cannot advert to Mr. Lee's account of the individual institutions in France, Italy, and Germany. Those who intend to visit them will take the book with them—and to others the account would not be very interesting. Mr. Lee has judiciously selected some clinical cases, illustrating the practice pursued at the different hospitals, and he has wound up the volume with an amusing account of animal magnetism and homœopathy—those precious effusions of German ideality, for which we refer our readers to the work itself. Mr. Lee is again on the Continent in search of knowledge, and will doubtless enrich the next edition with some valuable gleanings.

* Dict. de Médecine et Chirurgie Pratique. Art. Mercure.

